# Multi-year soundscape recordings and automated call detection reveals varied impact of moonlight on calling activity of neotropical forest katydids

**DOI:** 10.1098/rstb.2023.0110

**Published:** 2024-06-24

**Authors:** Laurel B. Symes, Shyam Madhusudhana, Sharon J. Martinson, Inga Geipel, Hannah M. ter Hofstede

**Affiliations:** ^1^ K. Lisa Yang Center for Conservation, Cornell University, Ithaca, NY 14853-0001, USA; ^2^ Smithsonian Tropical Research Institute, Luis Clement Avenue, Building 401 Tupper Ancon, Panama, Republic of Panama; ^3^ Centre for Marine Science and Technology, Curtin University, Perth, WA 6845, Australia; ^4^ Department of Fish, Wildlife & Conservation Biology, Colorado State University, Fort Collins, CO 80523, USA; ^5^ Active Perception Lab, Department of Engineering Management, University of Antwerp, 2020 Antwerpen, Belgium; ^6^ CoSys Lab, Faculty of Applied Engineering, University of Antwerp, 2020 Antwerpen, Belgium; ^7^ Flanders Make Strategic Research Centre, 3920 Lommel, Belgium; ^8^ Department of Integrative Biology, University of Windsor, Windsor, Ontario, Canada, N9B 3P4

**Keywords:** Orthoptera, moonlight, tropical biology

## Abstract

Night-time light can have profound ecological effects, even when the source is natural moonlight. The impacts of light can, however, vary substantially by taxon, habitat and geographical region. We used a custom machine learning model built with the Python package *Koogu* to investigate the *in situ* effects of moonlight on the calling activity of neotropical forest katydids over multiple years. We prioritised species with calls that were commonly detected in human annotated data, enabling us to evaluate model performance. We focused on eight species of katydids that the model identified with high precision (generally greater than 0.90) and moderate-to-high recall (minimum 0.35), ensuring that detections were generally correct and that many calls were detected. These results suggest that moonlight has modest effects on the amount of calling, with the magnitude and direction of effect varying by species: half of the species showed positive effects of light and half showed negative. These findings emphasize the importance of understanding natural history for anticipating how biological communities respond to moonlight. The methods applied in this project highlight the emerging opportunities for evaluating large quantities of data with machine learning models to address ecological questions over space and time.

This article is part of the theme issue ‘Towards a toolkit for global insect biodiversity monitoring’.

## 1. Introduction

Daily, monthly and seasonal light cycles are among the strongest temporal structuring forces in biology. Light cycles can influence plant phenology, the timing of animal activity, hibernation, reproduction, and other key ecological and physiological processes [1–4]. Moon illumination levels fluctuate by up to three orders of magnitude over the 29.5 day lunar cycle [5], exposing organisms to significant cyclic changes in night-time light levels. These fluctuations can have profound ecological and evolutionary consequences [1,6–8] . Understanding how nocturnal light levels impact ecological communities is important because it can highlight key relationships and responses that may be distorted by anthropogenic light, with possible conservation implications.

Variation in moonlight intensity can influence animal activity levels at night [1,9]. In some cases, these are direct responses to variation in light levels. For example, nocturnal species that forage visually may have greater foraging success on moonlit nights and forage for longer [7,10,11]. Indirect effects of light are also possible, such as when animals decrease their activity or shift locations on moonlit nights to minimize exposure to visually hunting predators [12–14]. Light-based changes in risk and reward can propagate across trophic levels in complex ways when food webs contain many interactions and animals that are both predators and prey [7,15]. In a meta-analysis of 59 nocturnal mammal species, Prugh and Golden [16] found that moonlight intensity had variable impacts on activity, even within closely related species groups and across trophic levels, with increased activity particularly among primates and comparatively strong activity suppression of many taxa in open habitats.

Insects are one of the most diverse taxonomic groups, with a central role in food webs and terrestrial ecological processes [17,18]. Light is known to have strong effects on insect behaviour and artificial light at night likely plays a role in insect population declines through impacts on navigation and other behaviours [19–22]. Light-based trapping provides an effective tool for capturing many species of insects. Light captures can, however, vary by orders of magnitude depending on the phase of the moon and amount of cloud cover ([23] and LB Symes, SJ Martinson, HM ter Hofstede 2016–2024, unpublished data). Whether the variation in light catch across the lunar cycle reflects true fluctuations in insect activity levels, or the impacts of light on insect navigation near artificial light sources is not fully understood [23,24]. Some studies have found that insect activity followed lunar cycles even when insects were surveyed using non-light based methods, suggesting a true relationship with moonlight levels [25–27]. Other studies have found that although light trap catches followed a lunar cycle, other sampling approaches did not [23,28]. The lack of information on insect responses reveals the challenges of evaluating insect activity, particularly *in situ* and in the dark.

Orthopterans and other singing insects provide a useful lens for studying behavioural responses to light because their acoustic signal production can be monitored non-invasively and without needing to trap them using light. In laboratory experiments, Botha *et al*. [29] found no impact of manipulated light levels on song rate or structure in the cricket *Teleogryllus oceanicus*, whereas Levy *et al*. [30] found that lifetime exposure to light affected circadian activity in the cricket *Gryllus bimaculatus*. In field studies, Gomez-Morales & Acevedo-Charry [31] examined moonlight impacts on the calling of orthopterans in Colombia, determining that cricket calling was unaffected by moon phase, but that there was a negative relationship between moon illumination and calling activity in all five sampled katydid species (or sonotypes, when animals could not be captured or identified). Lang *et al*. [32] studied katydid activity in older growth neotropical forest quantifying katydid captures at lights, katydid bycatch in mist nets (set for bats), and total sound levels in ambient forest recordings throughout the moon cycle. They found support for effects of light on activity, with more insects caught at light traps during nights near new moon, and a higher prevalence of katydids captured in mist nets during the darker half of the month (62% of the total captures after correction for sampling effort). The sound levels in the middle of the night in the forest were also approximately 10 dB higher during the new moon, a finding that suggests a lunar cycle of biological activity, although the broad frequency range in which sound was sampled makes it difficult to attribute the response to a particular taxonomic group. In a focal study of the neotropical forest katydid species *Docidocercus gigliotosi,* researchers found little impact of moon illumination on acoustic signalling, but strong impacts on the amount of vibrational signalling produced [33]. These results show that the response of orthopteran insects to light is highly variable and might be related to specific aspects of each species' natural history. For example, crickets often call from burrows within which they are protected from predators whereas katydids usually call from vegetation and could face greater risks from acoustically and visually orienting predators.

The goal of this study is to use data for multiple katydid species in the same environment at the same time to test the hypothesis that katydid calling rates are influenced by moonlight with the prediction that calling rate decreases with increasing light levels. To survey multiple taxa over multiple years (2019–2021), we used acoustic monitoring and a machine learning-based method to detect and classify signals. By using *in situ* recordings, we were able to study behaviour in the context of all its natural complexity, including the presence of a suite of predators, mates, competitors, and hiding and foraging opportunities. This approach allowed us to assess whether signalling of these katydid species varies with moonlight, and if so, whether this response is uniform or variable across species.

## 2. Methods

### (a) Acoustic recording

**Figure 1.**
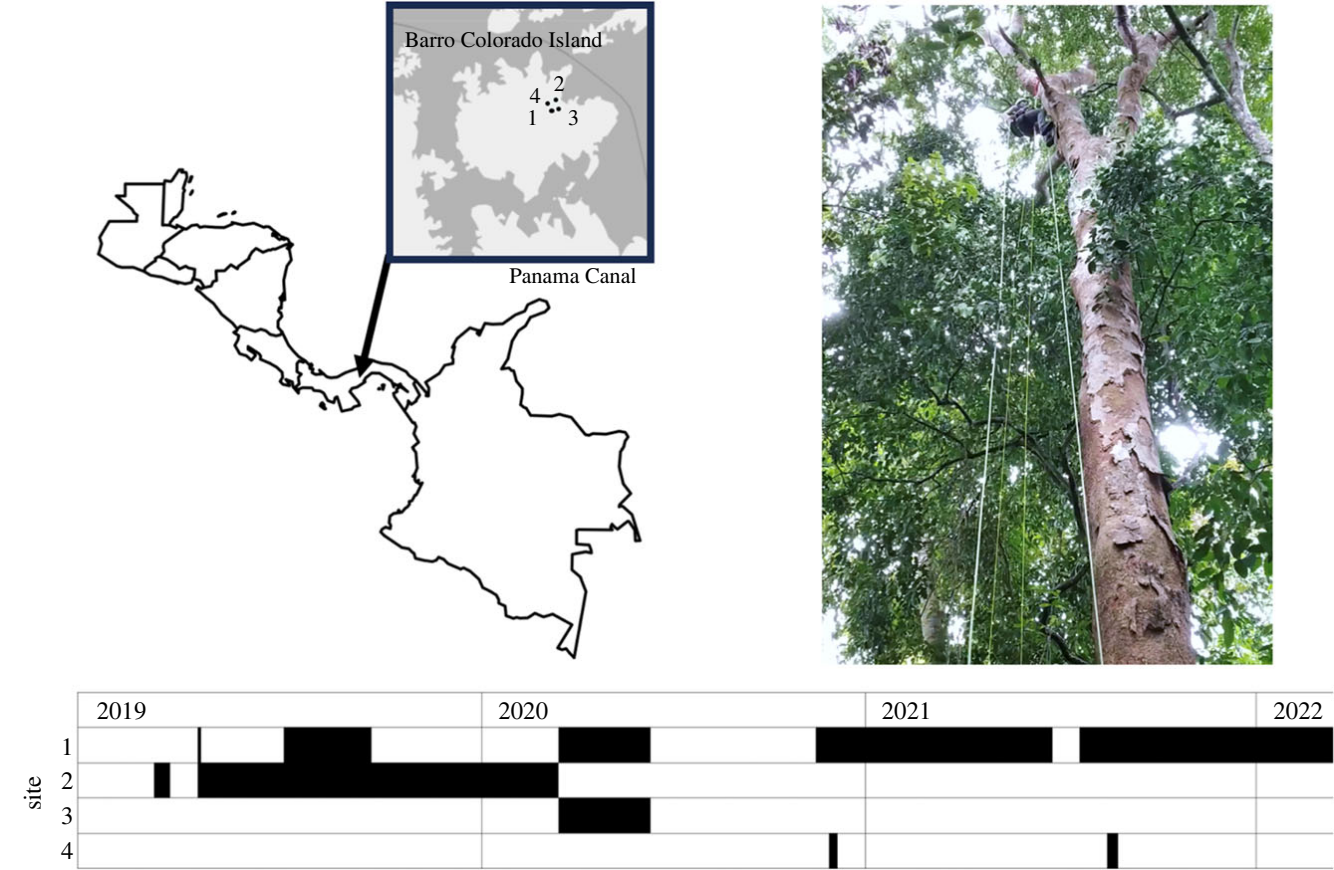
Recordings sites were located on Barro Colorado Island, an island in the Panama Canal (upper left map shows Panama and surrounding countries, inset map shows Barro Colorado Island within the Panama Canal and recording locations; coordinates given in electronic supplementary material, table S1). Maps were generated using R and Wikimapia. Recorders were installed in trees at a height of approximately 24 metres (upper right, showing the process of recorder installation). Recorders were operated intermittently at four recording sites (lower portion of figure, with black bars showing site-specific periods of operation; see electronic supplementary material, table S1 for dates and coordinates).

To determine how moonlight affects katydid calling activity, we collected multiyear soundscape recordings from Barro Colorado Island (BCI; 9°9′17N, 79°51′53W), Panamá, a site with very low impacts from anthropogenic light (figure 1; electronic supplementary material, table S1). This 15.6 km^2^ island is located in the Panama Canal and is mostly covered with primary and old secondary, semi-deciduous tropical moist forest [34,35]. Acoustic recordings were collected at a sampling rate of 96 kHz using Swift recording units that were built into a Pelican case (‘Rugged Swift’, K. Lisa Yang Center for Conservation Bioacoustics, Cornell University, US). Recordings were initially made at a gain of 33 dB, with gain increased to 35 dB on 4 June 2019. Subsequently, we determined that the electronic noise floor of the equipment determined the detection limit of the equipment at both gain settings, meaning that the higher gain setting did not result in detection of more distant signals.

Recording equipment was suspended at a height of 24 metres using pulley systems tethered to the branches of emergent trees (figure 1). The height of the recording units corresponded to the lower part of the forest canopy layer (average canopy height on BCI approx. 35 m; [36,37]). We recorded at this height because it is where moonlight levels would vary the most compared to the shaded understorey, making it more likely that we would detect changes in behaviour with light levels. We set the recording units to record for 10 min at the beginning of each hour from 18.00–06.00 (13 recordings per night). We collected recordings opportunistically from 30 January 2019 to 26 January 2022 (electronic supplementary material, table S1). Recording gaps were due to personnel being unavailable to service equipment, equipment failure and Covid-19 related site access restrictions.

### (b) Acoustic signal detection and classification

To evaluate the activity of different katydid species, we developed a convolutional neural network (CNN) model using *Koogu*, a Python-based machine learning toolbox [38]. We used a custom implementation of the quasi-DenseNet architecture [39] for identifying 31 species of katydids. We trained the model using a combination of annotated datasets comprising focal recordings [40] and soundscape recordings from BCI, and playback recordings wherein the higher-quality focal recordings were played back through vegetation to mimic environmental transmission effects. The full training set consisted of nearly 4500 calls, ranging from 20–680 exemplars per species. For full details of model development, see Madhusudhana *et al.* [41].

We assessed the trained CNN model's recognition performance characteristics using recordings from a separate test set [42]. The recordings were fed to the model as short (0.8 s) overlapped (0.6 s) segments. Contiguous segments producing detection scores above a threshold were combined using a post-processing algorithm to report a single detection. Comparing the reported detections to human-made annotations from the test set, we established two measures of model performance: precision and recall. For evaluating the rate of precision (the fraction of model identifications that were actually correct), we considered detections that overlapped with at least 60% of an annotation as true positives. For evaluating the recall rate (the fraction of annotated calls in the recording that were detected by the model), we considered that a call was successfully recalled when at least 50% of the human annotation was matched by one or more reported detections.

The model returned detections that corresponded to the detections made by humans, as well as apparent false positives that were not in the human annotated dataset. We inspected these apparent false positive detections carefully, including band-pass filtering the recording to the characteristic frequency of the species and inspecting the waveform to highlight temporal features. Filtering often revealed pulse timing that allowed us to confirm that the apparent false positives were in fact very faint signals with the temporal structure of the focal species. In some cases, however, even filtering and careful inspection failed to allow humans to confirm or reject a detection. Signals that could not be confirmed or rejected were typically faint signals that occurred at the correct frequency and approximate duration for the species, but where degradation obscured the temporal features used by human reviewers for identification. Finally, some of the apparent false positives were in fact false positives and represented detections of other sounds in the environment that could be clearly rejected by a human reviewer. Consequently, we report three metrics of model performance. The first metric is performance on the original human annotated data (table 1, Base). The second metric is performance after the human-confirmed detections were added to the test dataset (table 1, Base + Confirmed). The third metric essentially gives the model the benefit of the doubt and adds to the test data both the confirmed detections and any detections that could not be rejected by humans as false positives (table 1, Base + Unable to reject). The true performance of the model for a given species likely falls between the estimates provided by (Base + Confirmed) and (Base + Unable to reject), with the range between these performance metrics being relatively small in most species.

**Table 1.** Species-level precision and recall values for eight focal species and three model criteria. Precision is the proportion of detections that were true positives. Recall is the proportion of known focal events that were found by the model. Base, test set consists of human annotated calls; Base + Confirmed, test set of human annotations plus model detections confirmed by humans; Base + Unable to Reject, test set of human annotations, confirmed model detections, and model detections that could not be rejected as false positives (see Methods for additional description).

species	base		base + confirmed		base + unable to reject	
	precision	recall	precision	recall	precision	recall
*Acantheremus major*	0.010	0.418	0.730	0.544	1.000	0.560
*Anaulacomera furcate*	0.642	0.592	0.838	0.517	0.841	0.515
*Anaulacomera spatulate*	0.863	0.383	0.992	0.384	0.992	0.384
*Erioloides longinoi*	0.728	0.735	0.944	0.683	0.996	0.578
*Euceraia insignis*	0.767	0.404	1.000	0.380	1.000	0.370
*Montezumina bradleyi*	0.905	0.525	0.968	0.498	0.972	0.433
*Pristonotus tuberosus*	0.564	0.595	0.838	0.415	0.902	0.929
*Thamnobates subfalcate*	0.452	0.378	0.827	0.364	0.971	0.320

Across the three sets of metrics, precision and recall generally increased when incorporating events detected by the model and not the human, however, recall sometimes decreased slightly due to the post-processing step. Recall decreases could occur because human review of detections directed reviewers to additional calls that were then added to the test dataset, but these calls were not detected by the model with sufficient temporal coverage to be considered correct detections.

For this study, when using the model to subsequently process field recordings, we required high precision detections (with low false positive rates). We also required a moderately high rate of recall to ensure that we were capturing many of the calls present in the soundscape. We restricted the final species list to eight focal species that met the following criteria. Included species had a Base + Confirmed precision of at least 0.84 (greater than 0.9 for all but one species). These species also had at least 20 calls in the Base + Confirmed dataset and a recall of at least 0.3, although it was frequently higher (table 1). The detection threshold corresponding to these constraints was 0.95. While precision and recall varied by species, it remains possible to make intraspecific comparisons about detection rates as long as performance remains consistent across moon phases.

All field recordings were processed to determine the number of detections of each species at each site. In contrast to the test dataset, we used a segment overlap of 0.4 s (between successive segments) when processing these recordings since no post-processing of detections was performed. While the reduced segment advance boosted the processing throughput, it had little-to-no impact on recognition performance. The subsequent response variable is the number of detections (segments with scores above the threshold of 0.95) of the target signal within each 10 min recording.

### (c) Moon phase

We calculated the moonlight intensity for each 10 min recording using the R package *moonlit*, which accounts for the date, location of the site and whether the moon is waxing or waning [43].

### (d) Statistical analysis

We used model comparison to evaluate the impact of moon illumination on the calling activity of the eight focal katydid species. We created and compared three models, all modelling data with a zero-inflated distribution using the R package *glmmTMB* [42] (see analysis code in supplemental materials). For the first model, we included the number of detections per 10 min recording as the response variable; moon illumination, species, and the interaction of moon illumination and species as fixed effects; time block and site ID as random effects; and crossed random effects of species by site and species by time (reflecting the fact that site suitability could vary in ways that differ by species and that species may have different activity periods). For the second model, we removed just the interaction term between moon illumination and species to evaluate whether the interaction significantly improved model fit (i.e. species differed in their response to moonlight). For the third model, we used the same structure as the first model but removed the main effect of moon and the interaction between moon and species. We then calculated the difference in AIC values separating the three models [44].

## 3. Results

**Figure 2.**
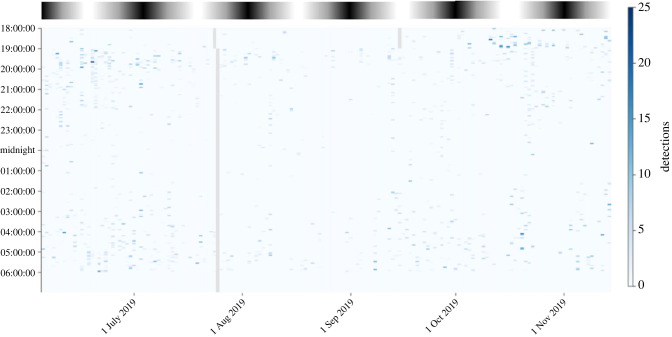
Detections of *Anaulacomera spatulata,* one of the eight focal species, at one location over a period of approximately five months. Moon phase is represented by the shaded bar at the top of the image, with bright intervals corresponding to full moon and dark intervals corresponding to new moon. For each hour, the associated 10 min recording is shown. The number of detections per unit time is represented with different grades of blue shading. The grey vertical line represents a day when the recorder was being serviced.

**Figure 3.**
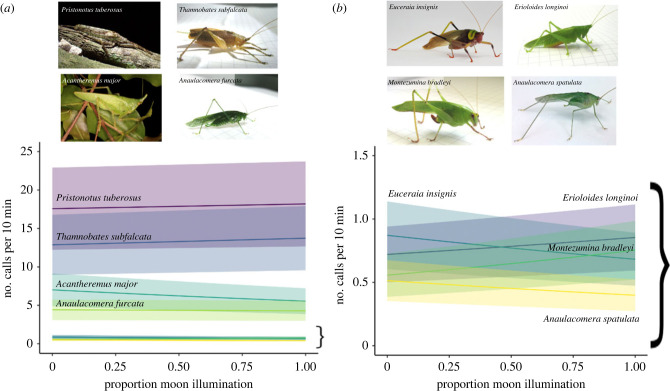
Predicted number of calls for eight katydid species as a function of moon illumination. All species (left panel) and inset of lowest calling rate species with expanded axis (right panel).

For all eight species, detections were common (figures 2 and 3), ranging from more than 8000 detections of *Anaulacomera spatulata* to 273 646 detections of *Thamnobates subfalcata* (table 3)*.* The best fit model (as indicated by the lowest AIC score and a very large dAIC with the next best fitting model) included moon illumination and the interaction between moon illumination and species as predictors (table 2). However, in most species, moonlight had modest impacts on the predicted number of calls (electronic supplementary material, table S1; table 3). This supports the idea that moonlight influences calling in these katydid species, but not in the same way for each species. In half of the species, the predicted number of calls decreased as the moon became fuller. In the remaining species, the pattern was reversed, with increased calling as the moon became fuller (figure 3). In most species, moonlight had modest impacts on the predicted number of calls (electronic supplementary material, table S2; table 3). The *R*^2^ value for the fixed effects of the model (species, moon and species by moon interaction) was 0.12 and varied substantially by species (table 4).

**Table 2.** Model comparison for the complete model, the model without the interaction term, and the reduced model (both moonlight and the interaction terms excluded).

model	AIC	dAIC	d.f.	weight
species + moonlight + (species * moonlight)	1180665.6	0	28	1
species + moonlight	1182755.8	2090	21	< 0.001
species	1182929.6	2264	20	< 0.001

**Table 3.** Calling patterns of the eight focal species. The total detections column reflects the aggregate count of detections across all recording intervals, sites and time periods. The new and full moon columns provide the anticipated number of detections per 10 min on full moon and new moon nights, predicted via the most strongly supported model. Percent change in calling activity shows the difference in predicted detections between new and full moon, calculated as 100*(new moon-full moon)/new moon. *R*^2^ indicates the species-level model fit.

species	subfamily	total detections	new moon (detections/10 min)	full moon (detections/10 min)	delta detections/10 min	% change	*R*^2^
*Acantheremus major*	Copiphorinae	254 996	7.02	5.54	1.48	21.1	0.13
*Anaulacomera furcate*	Phaneropterinae	54 351	4.42	4.42	0.00	0.0	0.09
*Anaulacomera spatulate*	Phaneropterinae	8309	0.51	0.40	0.12	22.5	0.01
*Erioloides longinoi*	Copiphorinae	15 441	0.72	0.85	−0.13	−18.6	0.15
*Euceraia insignis*	Phaneropterinae	13 539	0.87	0.68	0.19	21.7	0.00
*Montezumina bradleyi*	Phaneropterinae	10 377	0.56	0.75	−0.20	−35.4	0.02
*Pristonotus tuberosus*	Pseudophyllinae	262 316	17.58	18.20	−0.62	−3.5	0.11
*Thamnobates subfalcate*	Pseudophyllinae	273 646	12.88	13.74	−0.86	−6.7	0.15

## 4. Discussion

Automated detection and classification of insect calls provides exceptional power for evaluating a range of ecological questions. Through the examination of 892 975 detections of eight Neotropical forest katydid species, we were able to assess whether signalling activity changed with the amount of moon illumination. While we found support for the model that included moonlight and its interaction with species, the illumination of the moon explained modest amounts of the total variation in calling activity of these eight species. When moon illumination did impact calling activity, the relationship between calling and moon illumination was positive in some species and negative in others, indicating that the responses to moon illumination were not uniform across the different species (electronic supplementary material, table S2). However, a substantial portion of the total variation in detections was not explained by moonlight, time or site. Weather, predator activity and intraspecific interactions, among other factors, were likely to contribute to explaining the residual variation in detection rates.

Response to moonlight varied by species, but the response was not easily predicted from existing natural history information. Subfamily, body size and coloration did not predict whether relationships were positive or negative. In some species, there was a 20–30% difference in the number of calls present in the forest, depending on whether the moon was new or full. Variation in the number of calls detected is important to understand when interpreting relative detection rates between sites and years. The absolute change in number of calls produced, however, was often very small and it is difficult to know whether changes on the order of 0.2 calls/10 min translate to meaningful differences in mating behaviour or predation risk.

Moon illumination has dramatic impacts on the effectiveness of light trapping for katydids and on the activity levels of many other taxonomic groups [16,23,24]. The tendency of these neotropical forest katydids to call irrespective of moon phase emphasizes that they are continuing to advertise for mates across a range of light levels. Tropical forest katydids may show modest variation in calling activity with moon phase because their signalling strategy is already well-tuned to minimize conspicuousness to predators [45,46]. Alternatively, in many species, the adult lifespan may only be a few months and the cost to an individual of waiting weeks for more favourable light conditions may have greater fitness impacts and risk of mortality than calling when light conditions are unfavourable.

Like other sampling approaches, acoustic sampling comes with a set of advantages, constraints and considerations for interpretation. The data collected in this study were collected *in situ*, providing a large sample size and capturing behaviour in the full complexity of the tropical forest. However, the species that we focused on for this study produced signals that occurred relatively frequently (appearing often in human-annotated test datasets), and for which precision was high with substantial recall. It is possible that species that call more rarely or are less well-recognized by automated approaches may show greater sensitivity to moonlight.

Quantifying sensitivity to light, both natural and anthropogenic, is important for understanding the basic ecology of species, and also for managing habitat preservation, land use change and other conservation activities. In our study, we found minimal response in katydid calling as a function of moonlight in our forested habitat. In more open secondary-grown habitats in nearby Colombia, katydids did decrease calling activity in response to moonlight [31]. It is not entirely surprising that insect responses to light vary, even among related species in geographical proximity. Like larger taxa, the behavioural and physiological responses of insects to light likely depend on evolutionary history, contemporary interactions and an array of additional environmental factors, meaning that the responses of one species or community in one location may, or may not, generalize to responses of other species in other locations. To generate a synthetic understanding and predictive framework for how species respond to environmental conditions such as natural and artificial light, it is essential to survey a variety of species and environments. Understanding how light sensitivity varies geographically, taxonomically and by habitat type is especially important when seeking to understand how species and communities might respond to anthropogenic light [3].

Automated signal detection and classification can provide immense power for non-invasively surveying insects over space and time and for understanding ecosystems. Some researchers have previously developed approaches for classifying insects in focal recordings (e.g. [47,48]). However, few passive acoustic monitoring studies have attempted to resolve individual species or genera within the acoustic insect assemblage, with insect sounds sometimes approached as noise that make soundscape interpretation more difficult. Despite the challenges, there is a vast amount of information present within the insect chorus. Particularly for research on restoration impacts and land use change, insects provide valuable metrics [49]. While birds, mammals and other large taxa are more visible and acoustically distinctive to humans, they are often highly mobile, obscuring the relationship between local habitat modification and species-level changes [50]. By contrast, insect communities can and often do vary on a scale of metres, providing nuanced detail about how habitats are colonized and occupied and how they are similar or different across time and space.

The ability to use automated detection of insects as part of habitat monitoring is limited in part by the availability of insect sound in libraries such as Orthoptera Species Files [51]. While these collections are growing, many insect species have never been recorded, particularly in the tropics. As automated approaches continue to advance and produce large and detailed datasets, the ability to apply computational approaches to insect sound will continue to depend on the quality of field data and associated taxonomic work. If field biology and machine learning approaches are synergized, it will increase by orders of magnitude the ability to sample insect communities over space and time, with real consequences for basic biology and conservation.

## Data Availability

Raw detection data and statistical analysis code are provided as electronic supplemental material [52].
